# A Simple Spatial Working Memory and Attention Test on Paired Symbols Shows Developmental Deficits in Schizophrenia Patients

**DOI:** 10.1155/2013/130642

**Published:** 2013-11-20

**Authors:** Wei Song, Kai Zhang, Jinhua Sun, Lina Ma, Forrest Fabian Jesse, Xiaochun Teng, Ying Zhou, Hechen Bao, Shiqing Chen, Shuai Wang, Beimeng Yang, Xixia Chu, Wenhua Ding, Yasong Du, Zaohuo Cheng, Bin Wu, Shanguang Chen, Guang He, Lin He, Xiaoping Chen, Weidong Li

**Affiliations:** ^1^Bio-X Institutes, Key Laboratory for the Genetics of Developmental and Neuropsychiatric Disorders, Ministry of Education, Shanghai Jiao Tong University, Shanghai 200240, China; ^2^Wuxi Mental Health Center, Wuxi 214151, China; ^3^Shanghai Mental Health Center, Shanghai Jiao Tong University School of Medicine, Shanghai 200030, China; ^4^Department of Endocrinology and Metabolism, The First Affliliated Hospital, China Medical University, Shenyang 110001, China; ^5^National Key Laboratory of Human Factors Engineering, China Astronaut Research and Training Center, Beijing 100094, China

## Abstract

People with neuropsychiatric disorders such as schizophrenia often display deficits in spatial working memory and attention. Evaluating working memory and attention in schizophrenia patients is usually based on traditional tasks and the interviewer's judgment. We developed a simple Spatial Working Memory and Attention Test on Paired Symbols (SWAPS). It takes only several minutes to complete, comprising 101 trials for each subject. In this study, we tested 72 schizophrenia patients and 188 healthy volunteers in China. In a healthy control group with ages ranging from 12 to 60, the efficiency score (accuracy divided by reaction time) reached a peak in the 20–27 age range and then declined with increasing age. Importantly, schizophrenia patients failed to display this developmental trend in the same age range and adults had significant deficits compared to the control group. Our data suggests that this simple Spatial Working Memory and Attention Test on Paired Symbols can be a useful tool for studies of spatial working memory and attention in neuropsychiatric disorders.

## 1. Introduction

Studies have shown working memory [1–3] and attention [4, 5] deficits in individuals with schizophrenia. Working memory is a temporary storage facility that lasts for only seconds, accessible to conscious attention. It is fundamentally important as it underpins capacity for complex thought [6]. The visuospatial sketchpad theory proposes that the visual and spatial impression of a limited number of objects is temporarily stored, manipulated, and allows subselections to be created through the focus of attention [7]. Attention is the cognitive process of selecting some elements, from an environment (real or otherwise) while ignoring other elements and it is a precondition for exercising working memory. It determines what enters into working memory, and shifting attention can disrupt the contents of working memory. When presented with a shorter stimulus, the stimuli's entrance into working memory may be less complete [8].

Currently, tests for working memory include the n-back task [9] which is a continuous performance task commonly used to measure a part of working memory [10], the Sternberg Item Recognition Paradigm [11, 12], the Visual Patterns Test [13], the Spatial Working Memory task (SPWM) [14], and others [15–18]. Existing tests for attention include the Continuous Performance Test (CPT) [19, 20] and the Sustained Attention Test [21]. Some existing tests require extended periods of time to complete or require verbal knowledge, which may impede the subject's ability to complete the test due to age or education. With the objective of creating a simple test of working memory and attention for use in treatment of neuropsychiatric disorders, we developed a Spatial Working Memory and Attention Test on Paired Symbols (SWAPS). 

## 2. Materials and Methods

### 2.1. Subjects

The study tested 188 healthy volunteers from 12 to 60 years old. They were divided into 5 groups by age: a 10s age group ranging from 12 to 16 years old (14.2 ± 0.1, *n* = 43 (male = 23, female = 20)); 20s age group ranging from 20 to 27 years old (23.8 ± 0.2, *n* = 37 (male = 18, female = 19)); 30s age group ranging from 30 to 39 years old (33.7 ± 0.5, *n* = 41 (male = 21, female = 20)); 40s age group ranging from 40 to 49 years old, (45.5 ± 0.5, *n* = 35 (male = 17, female = 18)); 50s age group ranging from 50 to 60 years old (53.5 ± 0.5, *n* = 32 (male = 17, female = 15)). All schizophrenia patients were recruited from Shanghai Mental Health Center and Wuxi Mental Health Center. They had been diagnosed with schizophrenia according to the criteria set forth by the DSM-IV-TR publication, with the following exclusion criteria: alcohol or drug abuse or dependence, major medical or neurological illness, or noticeable lower intelligence as determined by at least two professional psychiatrists. A total of 72 schizophrenia patients currently undergoing treatment in these hospitals, ages 13 to 40, were divided into 3 groups according to age: a SZ10s group ranging from 13 to 19 years old (15.8 ± 0.2, *n* = 24 (male = 11, female = 13)); SZ20s group ranging from 20 to 29 years old (25.0 ± 0.5, *n* = 24 (male = 12, female = 12)); SZ30s group ranging from 30 to 40 years old (35.9 ± 0.6, *n* = 24 (male = 12, female = 12)). All subjects participated WITH their own free will and with informed consent. Subjects under age 18 also participated with parental consent. The study was approved by the Bio-Ethics Board of the Bio-X Institutes, Shanghai Jiao Tong University. 

### 2.2. SWAPS Test

**Figure 1:**
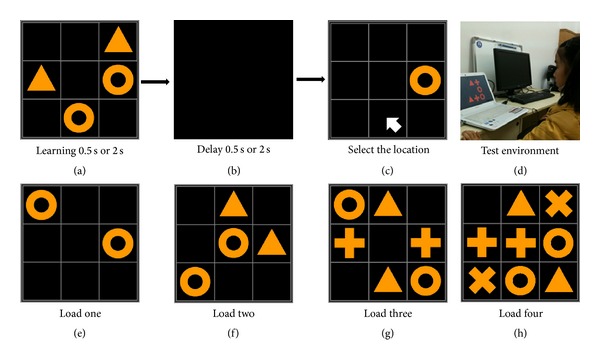
Example of a single trial and examples of 4 loads of difficulty. An example of a single trial ((a), (b), and (c)). The stimulus phase with learning time (Lt) of either 2 seconds or 0.5 seconds (a), the symbols then disappear during delay span (*D*) for either 2 seconds or 0.5 seconds (b). After the delay span, the participant is prompted to select the location of the missing part of the symbol pair with the pointing device (c). (d) shows a participant taking the test. (e)–(h) show examples of the 4 loads of difficulty. Difficulty load 1 is not used in results and exists only to provide a short practice exercise for the subject prior to the administration of the real test. Loads 2 to 4 are used to produce test results, and illustrate the number of paired symbols in each load.

The novel Spatial Working Memory and Attention Test on Paired Symbols (SWAPS) was inspired by the Chinese historical *Jiugongtu* (see Supplementary Figure 3 in Supplemetary Materials available online at http://dx.doi.org/10.1155/2013/130642) [22, 23]. *Jiugongtu* is well known in Chinese culture, making it a familiar spatial map for subjects. The SWAPS test uses the nine spaced *Jiugongtu* grid as a background but filled with paired symbols. SWAPS is a computer-administered test developed in the C# programming language. The test is composed of a visual two-dimensional grid plane presented to the subjects on a computer screen (Figure 1). The grid is segmented into nine regular squares, each of which might contain a symbol. There are 4 possible paired symbols that can be displayed in the grid. The subjects have been instructed to use a pointing device to select the grid location of the matching and missing symbol on the screen. The test randomly presents symbol pairs in different locations on the grid, for a learning time duration (Lt) of 0.5 seconds or 2 seconds (Figure 1(a)). The test then presents an empty screen for a duration of time which we call the delay span (*D*), which is 0.5 seconds or 2 seconds (Figure 1(b)), and then randomly presents only one of the symbols. The participants are expected to select the location of the missing one of the paired symbols (Figure 1(c)) or an “uncertain” button beside the grid. As the learning time Lt and delay span *D* can be 0.5 seconds or 2 seconds, this allows for 4 possible combinations. We record the reaction time of the participants beginning from when the symbol is presented during the memory recall phase (Figure 1(c)) and ending when the subject makes a choice with the pointing device. The percentage of correct choices (*C*) divided by reaction time (*R*) produces a score, calculated as the efficiency score (*C*/*R*). We also show the percentage of correct choices and reaction time results separately in Supplementary Figures 4–8 and Supplementary Tables 3–5. There are 4 levels of difficulty, which we call loads (1 to 4), where the load is the quantity of paired symbols presented on one grid. Load one has a single pair of symbols, load two has 2 symbol pairs, load three has 3 and load four has 4 (Figures 1(e)–1(h)).

### 2.3. The Testing Procedure

Subjects are first given an introduction to the test. An identifying number, their gender and their age are then recorded. The SWAPS test consists of 101 trials with the test lasting 7 minutes on average. The first 5 trials are all load one difficulty and are not used in the result. They exist to provide a short practice exercise for the subject prior to the administration of the real test. The next 96 trials are randomly arranged, consisting of 32 trials of all combinations of Lt (0.5 seconds or 2 seconds) and *D* (0.5 seconds and 2 seconds) per load, for difficulty loads two through four.

### 2.4. Statistical Analysis

All data were analyzed with Statview software. Results are expressed as mean ± SEM. All error bars represent standard error of the mean (SEM). ANOVA analyses and Fisher's PLSD [24] were used for statistical comparisons between groups as described in the results section or in figure legends. *P* < 0.05 indicates significant difference between groups.

## 3. Results

### 3.1. SWAPS Tests Showed a Developmental Peak of Spatial Working Memory and Attention in the 20–27 Age Range in the Healthy Control Group

**Figure 2:**
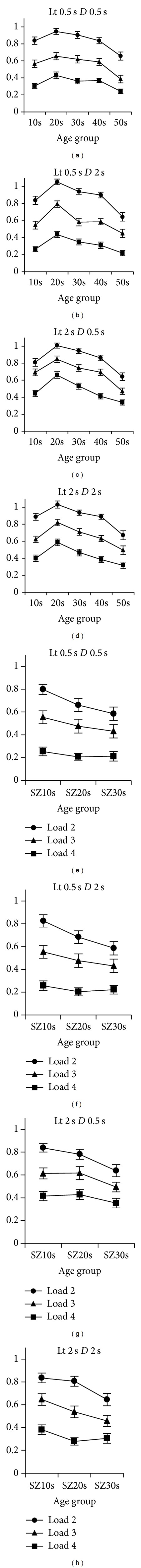
Comparison of schizophrenic and healthy groups. SWAPS showed the highest score in the age 20–27 healthy control group (group 20s) but not in schizophrenia patients ages 20–29 (group SZ20s). (a–d) show the accuracy per second in 5 healthy control age groups (10s, 20s, 30s, 40s, and 50s), at different difficulty loads and with different combinations of learning time and delay span. (e–h) show the accuracy per second for 3 age groups in schizophrenia patients (SZ10s, SZ20s, SZ30s), under the same learning time and delay span conditions as the healthy control groups. Lt: learning time (0.5 seconds or 2 seconds); *D*: delay span (0.5 seconds or 2 seconds).

We first used SWAPS to test normal healthy people. The control group was composed of 188 healthy volunteers from 12 to 60 years old. Age may be a factor influencing both accuracy and reaction time in completing SWAPS [25, 26], so participants were divided into groups by age as 10s, 20s, 30s, 40s,  and 50s. Compared to the 10s age group, the 20s group showed significantly better performance in almost all combinations of load level, learning time and delay span (Figure 2, Supplementary Table 1). SWAPS showed the development of spatial working memory and attention reached a peak in the 20s group and then declined with increasing age. With a shorter learning time of 0.5 seconds which requires closer attention, the result showed significantly better performance in the 20s age group (Figures 2(a) and 2(b)). Detailed statistical results are shown in Supplementary Table 1.

### 3.2. Schizophrenia Patients Showed No SWAPS Improvement with Age When Comparing SZ10s to SZ20s

Schizophrenia patients were then tested with SWAPS. Seventy-two schizophrenia patients 13 to 40 years old were divided into 3 groups according to age: SZ10s, SZ20s and SZ30s. Interestingly, schizophrenia patients failed to display maturation of spatial working memory and attention capacity in ages 20–29 compared to ages 13–19 (Figures 2(e)–2(h), Supplementary Table 1).

### 3.3. Schizophrenia Patients Displayed SWAPS Score Deficits in Adults but Not in Adolescents

**Figure 3:**
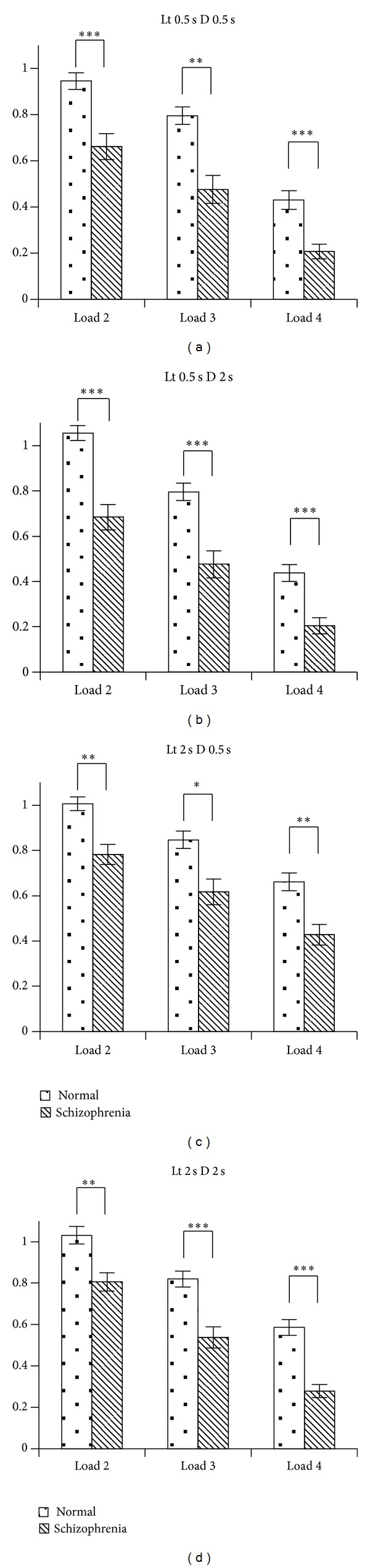
Comparative results of SZ20s and 20 s age groups. a–d show the accuracy per second of SZ20s and 20s age groups, at the different loads of difficulty with combinations of different learning time and delay span. *P < 0.05; **P < 0.01;^∗∗∗^ *P* < 0.0001.

Compared to the 20s control group, SZ20s schizophrenia patients displayed a significantly lower test score (Figure 3). The SZ30s group in schizophrenia patients also showed significant deficits in test scores under all combinations compared to the 30s control group (Supplementary Figure 2). However, SZ10s schizophrenia patients did not show a difference (under all test conditions) compared to the 10s control group (Supplementary Figure 1). Detailed comparative results of schizophrenia and control groups are shown in Supplementary Tables 1 and 2.

## 4. Discussion

Difficulty loads of symbols, stimulus duration (learning time), and delay span are important for measuring working memory and attention. In order to guide the development of SWAPS, we conducted a preliminary study in 31 healthy subjects with ages ranging from 18 to 24 years old. The preliminary study had random learning times (0–5 seconds) and delay spans (0–5 seconds) (Supplementary Figure 9). The first versions of the test needed to determine the most appropriate Lt and *D* to show differentiation between loads and we use correct responses to analyze because it is more sensitive within age groups. Supplementary Figure 9(a) shows the variation of correct responses through difficulty loads two, three and four, presenting evidence that the design of the test can discriminate between difficulty loads. We found that *D* of up to 2 seconds had the same results as *D* of up to 5 seconds (Supplementary Figure 9(b)). The added time did not increase accuracy in responses, and so we shortened *D* in our test design to 2 seconds. We found that Lt shows the largest differences between loads when Lt has a value of between 0 and 2 seconds, and when Lt exceeds 2 seconds the test results for all loads of difficulty begin to converge toward a higher percentage of correct response (Supplementary Figure 9(c)). Learning times of 0–2 seconds and delay spans below 2 seconds showed good results and could shorten the testing time. We use values of 0.5 seconds and 2 seconds [27, 28] for both learning time and delay span.

In the SWAPS test, observers focus their attention on the locations (spatial locations) and features (different symbols). A shorter learning time Lt of 0.5 seconds requires better attention. A longer delay *D* of 2 seconds requires better working memory, In the most challenging condition of Lt = 0.5 seconds, *D* = 2 seconds and load 4, the healthy control showed the lowest score, and 20 s control group showed peak score compared to other age groups. In contrast, the SZ20s schizophrenia group did not show the peak score when compared to the SZ10s and SZ30s groups.

Both accuracy and reaction time are important variables which reflect the memory and attention capacity of subjects who take the SWAPS test. We used an efficiency score (percentage of correct choice divided by average reaction time) [29] to display a simple result for working memory and attention. The schizophrenia group SZ20s showed more deficits in accuracy than reaction time compared to the 20s twenties age range control group (Supplementary Figure 7), and the SZ30s schizophrenia group showed greater deficits in reaction time than accuracy compared to the 30s thirties age range control group in thirties (Supplementary Figure 8). Using the efficiency score, results revealed clearer deficits in schizophrenia patients compared to their counterparts in the twenties and thirties age range. In other studies an inverse efficiency score is sometimes used [30]; however, because in some circumstances the *Q* score result of the SWAPS test can be 0, we use an efficiency score to quantify results. 

Cognitive abilities rise steeply from infancy to young adulthood and then are either maintained or decline to old age [31]. Brain activity studies have shown that healthy young adults develop better neurocognitive ability including working memory and attention [32, 33]. The SWAPS test has shown a plausible developmental pattern in healthy controls especially in difficulty load three and four, which developed well in the 20s group from the 10s group and then decline with increasing age. The more interesting finding in this study is that schizophrenia patients showed no working memory and attention improvement with age when comparing SZ10s to SZ20s. Is it because the developmental process of people with schizophrenia is halted that the spatial working memory and attention of the patients becomes worse in their twenties? To discover the answer to this, we may need to test more finely subdivided age groups both in control and in schizophrenia patients. More importantly, we may need to do a longitudinal study of SWAP tests from childhood to early adulthood for patients [34, 35].

A limitation of this study is that the schizophrenic group was comprised of patients who were diagnosed as schizophrenic and currently undergoing various treatments for schizophrenia. These treatments might influence the performance of spatial working memory and attention in patients. Besides, the age of onset, the course of disease, and education of participants maybe the influence factors, and we made a table (Supplementary Table 6) to show these detailed information. We must treat the conclusion with caution as we cannot exclude the effect of treatment in the current study. Because of these limitations, it is worth testing first-episode schizophrenia patients to confirm SWAPS test results in future studies. 

The studies reported the differences in visuospatial processing between males and females [36]. Here, gender was not a dominant factor affecting the score of SWAPS test (Supplementary Table 7). 

The test is simple, automatic, and results can be interpreted at the end of the test. Many people can simultaneously take the test. It does not burden the test participant more than requiring a few minutes of time looking at a computer screen. SWAPS was developed with the goal of creating a simple test of working memory and attention for use in clinical studies in China. However, it can be easily adapted by people worldwide as it uses simple symbols. The symbols and duration time also can be easily modified by the investigator for the purposes of their own studies. This short test of attention and spatial working memory may be a useful tool in studying other mental illness such as attention deficit hyperactivity disorder, major depressive disorders, and bipolar disorder. It may also be used as an objective indicator in determining effects of treatment for neuropsychiatric disorders. Moreover, SWAPS can be combined with fMRI, PET, or EEG methodologies in future studies. As the test is computer-based, it can easily be integrated into many situations and resulting data can be analyzed automatically.

## 5. Conclusion

SWAPS showed developmental maturation of spatial working memory and attention in ages 20–27 which then declined with increasing age. Schizophrenia patients failed to display a developmental peak of those cognitive abilities in ages 20–29 and had significant deficits compared to control groups in adults. Our data suggests that this simple SWAPS test can be a useful tool for studies of spatial working memory and attention in neuropsychiatric disorders.

## Supplementary Material

The novel Spatial Working Memory and Attention Test on Paired Symbols (SWAPS) was inspired by the Chinese historical jiugongtu (Supplementary Fig. 3).
SZ10s schizophrenia patients did not show a difference (under all test conditions) compared to the 10s control group (Supplementary Fig. 1). The SZ30s group in schizophrenia patients showed significant deficits in test scores under all combinations compared to the 30s control group (Supplementary Fig. 2). Detailed comparative results of schizophrenia and control groups are shown in Supplementary Table 1 and Table 2.
The percentage of correct choices and reaction time of SWAPS test were showed in supplementary figures 4-8 and supplementary tables 3-5.
Difficulty loads of symbols, stimulus duration (learning time) and delay span are important for measuring working memory and attention. In order to guide the development of SWAPS, we conducted a preliminary study in 31 healthy subjects with ages ranging from 18 to 24 years old. The preliminary study had random learning times (0-5 seconds) and delay spans (0-5 seconds) (Supplementary Fig 9).
The age of onset, the course of disease, and education of participants maybe the influence factors for SWAPS and we made a table (Supplementary Table 6) to show these detailed information.
The studies reported the differences in visuospatial processing between males and females. Here, gender was not a dominant factor affecting the score of SWAPS test (Supplementary Table 7).

## Abbreviations

SWAPS: Spatial Working Memory and Attention Test of Paired Symbols
*R*: Reaction time
*C*: Percentage of correct choices
Lt: Learning time
*D*: Delay span.
